# Anti-Cyclic Citrullinated Peptide Antibody Is Associated with Interstitial Lung Disease in Patients with Rheumatoid Arthritis

**DOI:** 10.1371/journal.pone.0092449

**Published:** 2014-04-17

**Authors:** Yufeng Yin, Di Liang, Lidan Zhao, Yang Li, Wei Liu, Yan Ren, Yongzhe Li, Xiaofeng Zeng, Fengchun Zhang, Fulin Tang, Guangliang Shan, Xuan Zhang

**Affiliations:** 1 Department of Rheumatology, Peking Union Medical College Hospital, Chinese Academy of Medical Sciences and Peking Union Medical College, Beijing, China; 2 Department of Radiology, Peking Union Medical College Hospital, Chinese Academy of Medical Sciences and Peking Union Medical College, Beijing, China; 3 Department of Epidemiology, Institute of Basic Medical Sciences, Chinese Academy of Medical Sciences and Peking Union Medical College, Beijing, China; Penn State University, United States of America

## Abstract

**Objective:**

Patients with rheumatoid arthritis (RA) are at risk to develop RA-associated interstitial lung disease (RA-ILD). This retrospective study aimed to investigate the potential association of the positivity of serum anti-cyclic citrullinated peptide antibody (anti-CCP2) and rheumatoid factor (RF) with RA-ILD in RA patients.

**Methods:**

A total of 285 RA patients were recruited at the inpatient service of Peking Union Medical College Hospital in China between 2004 and 2013. Individual patients were evaluated for the evidence of ILD. The concentrations of serum anti-CCP2 and RF in individual patients were measured. The potential risk factors for ILD in RA patients were assessed by univariate and multivariate models.

**Results:**

There were 71 RA patients with RA-ILD, accounting for 24.9% in this population. The positive rates of anti-CCP2 and RF in the patients with RA-ILD were significantly higher than that in the patients with RA-only (88.7% vs. 67.3%, p<0.001; 84.5% vs. 70.6%, p = 0.02, respectively). Univariate and multivariate logistic regression analysis revealed that RA patients with positive serum anti-CCP2, but not RF, were associated with an increased risk of ILD (crude odds ratio [cOR] 3.83, 95% confidence interval [CI] 1.74–8.43, p<0.001; adjusted odds ratio [aOR] 3.50, 95% CI 1.52–8.04, p<0.001).

**Conclusion:**

Our findings suggest that positive serum anti-CCP2, but not RF, may be associated with RA-ILD in RA patients.

## Introduction

Patients with rheumatoid arthritis (RA) display high levels of autoantibodies as well as extra-articular manifestations, such as interstitial lung disease (ILD) [1]–[3]. The RA-related interstitial lung disease (RA-ILD) occurs in nearly 7–10% of the RA patients, and often is associated with a poor prognosis [4], [5]. Therefore, the discovery of risk factors contributing to the development of ILD will be of great significance in the prevention and intervention of patients with RA-ILD.

Autoantibodies are valuable biomarkers for the diagnosis of RA and extra-articular manifestations. Antibodies against cyclic citrullinated peptides (anti-CCP2) and rheumatoid factor (RF) have been identified in patients with RA [6]. Previous studies have shown that the specificity and sensitivity of anti-CCP2 detection for the diagnosis of RA are 96–99% and 47–88% respectively, dependent on the characteristics of the RA population [6], [7]. Anti-CCP2 antibodies may be implicated in the pathogenesis of RA and are valuable for evaluating the erosive or non-erosive progression of articular injury in RA patients [8], [9]. In addition, anti-CCP2 antibodies have been shown to be highly specific or independently associated with the development of extra-articular manifestations, including ischemic heart disease [10], type 1 diabetes mellitus [11], serositis [12], and subclinical atherosclerosis in patients with RA [13]. RF is another autoantibody most commonly detected in RA [1], [14]. Detection of both anti-CCP2 and RF has additional values for the early diagnosis of RA, particularly for those with RA at early stage of the disease process [15].

However, the potential association of anti-CCP2 and RF with the development of ILD in RA patients remains controversial [16]–[18]. There is little information about whether anti-CCP2 antibodies or RF are associated with ILD in RA patients. In this study, we tested the levels of serum anti-CCP2 and RF in 285 patients with RA and analyzed the potential factors that were correlated with ILD in this population.

## Methods

### Ethics statement

The experimental protocol was approved by the Institute Review Board of Peking Union Medical College Hospital. All patients provide their written informed consent to participate in this study.

### Study population

This study was approved by the Institute Review Board of Peking Union Medical College Hospital. A total of 285 patients with RA were recruited at the inpatient service of the Department of Rheumatology of Peking Union Medical College Hospital from January 2004 to October 2013. All patients fulfilled the criteria for the diagnosis of RA revised by the American College of Rheumatology (ACR) in 1987 [1]. Patients with RA-ILD were diagnosed by the presence of typical features in the lung by high-resolution computerized tomography (HRCT). The chest HRCT scans were evaluated by an expert radiologist in a blinded manner. According to the consensus for idiopathic interstitial pneumonias of the American Thoracic Society/European Respiratory Society (ATS/ERS) [19], the features of HRCT included irregular linear or reticular opacities, ground-glass opacities, consolidation, honeycombing, septal thickening, and traction bronchiectasis or bronchiolectasis. The disease activity of individual patients was evaluated by disease activity score in 28 joints (DAS28) [20], [21]. Individuals were excluded if she/he had a history of ILD before the diagnosis of RA, other chronic lung diseases or incomplete medical record.

### Clinical assessment

The demographic and clinical data of individual patients were retrospectively reviewed. These data included age, gender, disease duration, and cigarette smoking, co-existent autoimmune diseases, such as systemic lupus erythematosus (SLE), polymyositis/dermatomyositis (PM/DM), systemic sclerosis (SSc) and Sjögren's syndrome. The disease duration was defined from the onset of joint swelling and/or tenderness. Individuals with previous history of treatment with biological or general disease-modifying anti-rheumatic drugs (DMARDs) and corticosteroids for more than three months were recorded.

Blood samples were obtained from individual patients when they first visited to our institution. The positivity for serum anti-CCP2 (≥25 U/ml) and RF (≥15 IU/ml) in these patients was determined by enzyme linked immunosorbent assay (ELISA) using the specific kit (Euroimmun, Lübeck, Germany) and nephelometry method (Behring, Germany), respectively. The concentrations of serum C-reactive protein (CRP) as well as the value of erythrocyte sedimentation rate (ESR) in individual patients were routinely examined.

### Statistical analyses

All values are expressed as the mean ± SD or median with interquartile range (IQR) for normally and non-normally distributed data, respectively. The difference in individual variables between the RA-ILD and RA-only groups was analyzed by univariate model. Comparison of continuous data was performed using independent Student's t and Mann-Whitney non-parametric U test for normal and non-normal data respectively, and of categorical data using the Pearson Chi-square test or Fisher's exact test. The stratification analyses for age and disease duration were conducted by Cochran-Mantel-Haenszel Chi-square test. The relationship of individual variables with RA-ILD was analyzed using a multivariate logistic regression model with stepwise selected variables. The collinearity of variables was assessed and explained by variance inflation factor (VIF) greater than 4.0 and tolerance less than 0.25 [22]. All statistical analyses were performed using the SPSS software (version 17.0, SPSS Inc., Chicago, IL, USA) and a two-tailed P-value of less than 0.05 was considered statistically significant.

## Results

### Patients and clinical characteristics

To determine the potential risk factors associated with ILD, a total of 285 patients with RA were recruited. Their demographic and clinical characteristics are summarized in **Table 1**. There were 71 patients with ILD, accounting for 24.9% in this population. The RA-ILD patients were significantly older and had significantly longer duration than that of the RA-only group of patients. However, there was no significant difference in the distribution of gender and in the percentages of smokers between the RA-only and RA-ILD groups of patients.

**Table 1:** The demographic and clinical characteristics of patients.

Characteristics	All patients	RA-ILD	RA-only	p-value
Patients, n (%)	285 (100)	71 (24.9)	214 (75.1)	—
Age, mean (SD), years	51.7 (13.4)	58.3 (11.2)	49.5 (13.4)	<0.001
Female, n (%)	211 (74.0)	50 (70.4)	161 (75.2)	0.44
Disease duration, median (IQR), years	5.0 (1.0–12.0)	9.0 (2.0–18.0)	4.0 (1.0–10.1)	0.003
Cigarette smoking, n (%)	59 (20.7)	18 (25.4)	41 (19.2)	0.31
Complication With other CTD*, n (%)	61 (21.4)	15 (21.1)	46 (21.5)	1.0
Laboratory test				
Anti-CCP2, n (%)	207 (72.6)	63 (88.7)	144 (67.3)	<0.001
RF, n (%)	211 (74.0)	60 (84.5)	151 (70.6)	0.02
CRP, mean (SD), mg/dL	40.2 (56.3)	42.6 (75.4)	39.4 (48.5)	0.68
ESR, mean (SD), mm/h	56.2 (34.5)	56.6 (33.2)	56.1 (35.0)	0.93
DAS28, mean (SD)	5.4 (1.7)	5.2 (1.8)	5.5 (1.7)	0.13
Treatment history, n (%)				
Prednisone	234 (82.1)	58 (81.7)	176 (82.2)	1.0
Methotrexate	180 (63.2)	38 (53.5)	142 (66.4)	0.07
Biological DMARD	38 (13.3)	6 (8.5)	32 (15.0)	0.23
Chemical DMARD	227 (79.6)	52 (73.2)	175 (81.8)	0.13

RA-ILD: rheumatoid arthritis-associated interstitial lung disease; RA-only: rheumatoid arthritis without interstitial lung disease; IQR: interquartile range; CTD: autoimmune disease; CRP: C-reactive protein; ESR: erythrocyte sedimentation rate; DAS28: disease activity score with 28 joints;

*Other CTD included systemic lupus erythematosus, polymyositis/dermatomyositis, systemic scleroderma and Sjögren's syndrome.

There was no significant difference in the percentages of complications and treatment history between these two groups of patients. Both groups of patients had similar levels of disease severity, the concentrations of serum CRP and ESR. There were 61 (21.4%) patients who had at least one other connective tissue disease (CTD), including 7 patients with SSc, 41 Sjögren's Syndrome, 4 PM/DM, and 16 SLE. There were 234 (82.1%), 180 (63.2%), 38 (13.3%) and 227 (79.6%) of patients receiving medication of prednisone, methotrexate, biological DMARD, or chemical DMARD, respectively (**Table 1**).

### HRCT features and anti-CCP2 and RF status

In 71 patients with RA-ILD, HRCT scans of the lung revealed 48 cases with irregular line or reticular opacities, 22 cases with ground-glass attenuation, 26 cases with basal consolidation, 10 patients with honey-combing, 27 patients with septal thickening and 12 patients with traction bronchiectasis or bronchiolectasis. However, these HRCT characteristics were not significantly associated with the positivity of anti-CCP2 and RF in these patients (**Table 2**).

**Table 2:** The relationship between HRCT features and antibody status of anti-CCP2 and RF in patients with RA-ILD.

HRCT feature	Anti-CCP2 (+)	Anti-CCP2 (−)	p	RF (+)	RF (−)	p
Irregular line/reticular opacities, n (%)	43 (68.3)	5 (62.5)	0.71	39 (65.0)	9 (81.8)	0.49
Ground-glass attenuation, n (%)	21 (33.3)	1 (12.5)	0.42	20 (33.3)	2 (18.2)	0.48
Basal consolidation, n (%)	23 (36.5)	3 (37.5)	1.0	20 (33.3)	6 (54.5)	0.19
Honey-combing, n (%)	8 (12.7)	2 (25.0)	0.31	9 (15.0)	1 (9.1)	1.0
Septal thickening, n (%)	24 (38.1)	3 (37.5)	1.0	24 (40.0)	3 (27.3)	0.52
Traction bronchiectasis/bronchiolectasis, n (%)	9 (14.3)	3 (37.5)	0.13	10 (16.7)	2 (18.2)	1.0

### The relationship between RA-ILD and anti-CCP2 or RF

Characterization of the levels of serum anti-CCP2 and RF revealed that 207 (72.6%) serum samples were positive for anti-CCP2 and 211 (74.0%) were positive for RF in this population. Both the positivity rates of anti-CCP2 and RF were significantly higher (p<0.001 and p = 0.02 respectively) in the group of RA-ILD than RA-only (**Table 1**). Cochran-Mantel-Haenszel stratification analyses indicated that the percentages of anti-CCP2 positivity were significantly associated with older age and longer disease duration (OR_M-H_ 3.3, 95% CI 1.5–7.1, p = 0.003 and OR_M-H_ 3.9, 95% CI 1.7–8.7, p = 0.001, respectively) (**Table 3**).

**Table 3:** Stratification analyses of the association of anti-CCP2 with ILD in RA patients.

	RA-ILD	RA-only	OR_M-H_ (CI 95%)	p
Age				
<46 years, n (%)	14 (15.1)	79 (84.9)		
47–58 years, n (%)	19 (19.4)	79 (80.6)	3.3 (1.5–7.1)	0.003
>59 years, n (%)	38 (40.4)	56 (59.6)		
Disease duration				
<1.9 years, n (%)	17 (20.5)	66 (79.5)		
2.0–9.9 years, n (%)	19 (18.6)	83 (81.4)	3.9 (1.7–8.7)	0.001
>10.0 years, n (%)	35 (35.0)	65 (65.0)		

OR_M-H_: Mantel-Haenszel odds ratio. 95% CI: 95% confidence interval. Age and disease duration of all patients were divided into three equal groups.

### Univariate and multivariate logistic regression analysis

Univariate logistic regression analysis revealed that age and disease duration were risk factors for RA-ILD (crude odds ratio [cOR] 1.06, 95% CI 1.03–1.09, p<0.001; and cOR 1.04, 95% CI 1.01–1.07, p<0.001 respectively). Treatment with methotrexate (MTX) or other chemical DMARDs was not significantly associated with RA-ILD (cOR 0.58, 95% CI 0.34–1.01, p = 0.05, and cOR 0.61, 95% CI 0.32–1.14, p = 0.12; respectively). The positivity of anti-CCP2 and RF was significantly associated with ILD in RA patients (cOR 3.83; 95% CI 1.74–8.43, p<0.001; and cOR 2.28; 95% CI 1.12–4.61, p = 0.02 respectively). Further stratification of low, moderate and high levels of RF indicated that only high levels of serum RF (≥364.0 IU/ml) were associated with ILD in RA patients (p = 0.02). After adjustment of age and disease duration together with the absence of collinearity (VIF and tolerance were 1.03 and 0.97 respectively) and other related co-variables (**Table 4**), the positivity of anti-CCP2 remained a risk factor for ILD in RA patients (adjusted odds ratio [aOR] 3.50; 95% CI 1.52–8.04, p<0.001), regardless of the level of anti-CCP. However, after adjusting confounding factors including gender, age, disease duration, smoking, medications and other CTD complication, there was no significant association between any level of low, moderate and high positivity of RF and RA-ILD in this population (**Table 4**).

**Table 4:** Univariate and multivariate analyses of the associations of anti-CCP2 positivity with the RA-ILD.

	Univariate associations			Multivariate associations		
	β	cOR (95% CI)	p	β	aOR (95% CI)	p
Age	0.06	1.06 (1.03–1.09)	<0.001	0.05	1.06 (1.03–1.08)	<0.001
Disease duration	0.04	1.04 (1.01–1.07)	<0.001	0.04	1.04 (1.01–1.07)	0.02
Anti-CCP2						
Seropositive	1.34	3.83 (1.74–8.43)	<0.001	1.25	3.50 (1.52–8.04)	<0.001
Low positive	1.27	3.57 (1.46–8.76)	0.01	1.24	3.46 (1.35–8.89)	0.01
Moderate positive	1.54	4.67 (1.93–11.29)	0.00	1.44	4.22 (1.63–10.90)	0.003
High positive	1.20	3.32 (1.35–8.20)	0.01	1.08	2.96 (1.14–7.67)	0.03
RF						
Seropositive	0.82	2.28 (1.12–4.61)	0.02	−0.04	0.96 (0.38–2.44)	0.93
Low positive	0.74	2.10 (0.91–4.86)	0.08	0.38	1.46 (0.54–3.94)	0.45
Moderate positive	0.75	2.12 (0.93–4.82)	0.07	0.25	1.28 (0.45–3.66)	0.65
High positive	0.97	2.63 (1.16–5.93)	0.02	0.66	1.93 (0.68–5.47)	0.21

Univariate and multivariate associations of anti-CCP2 with the occurrence of RA-ILD was conducted by logistic regression with a backward stepwise model. Covariates in multivariate model included gender, age, disease duration, smoking, medication, and other CTD complication. β: partial regression coefficient; DMARDs: disease-modifying antirheumatic drugs; Anti-CCP2: anti-citrullinated peptide antibody; COR: crude odds ratio; AOR: adjusted odds ratio; 95% CI: 95% confidence interval. Three equal groups of low, moderate and high positive anti-CCP2 were defined as 25.0≤anti-CCP2<271.3 U/ml, 271.3≤anti-CCP2<941.2 U/ml and anti-CCP2≥941.2 U/ml, respectively. Three equal groups of low, moderate and high positive RF were defined as 15.0≤RF<114.0 IU/ml, 114.0≤RF<364.0 IU/ml and RF≥364.0 IU/ml, respectively.

## Discussion

The pulmonary manifestations in RA patients was firstly reported half a century ago [23] and affect 10 to 20% of RA patients, which is associated with increased mortality [24]. The ILD has been recognized as the most common complication in the lung of RA patients. In this retrospective study, we evaluated the potential factors that contributed to ILD in RA patients. We found that 24.9% RA patients suffered with ILD, which was higher than that reported [3], [4]. The high rate of patients with ILD may be due to long disease duration in a large proportion of our RA patients. Indeed, we found that RA patients with ILD were significantly older and had longer duration than those patients without ILD in this population. Alternatively, the high rate of patients with ILD may stem from unique genetic background.

Some other risk factors for RA-ILD should also be considered in multivariate analysis of the associations of anti-CCP2 positivity with the RA-ILD. Firstly, a previous study has showed that ILD is a severe clinical entity presented in many connective tissue diseases [25]. However, anti-CCP2 has been demonstrated to be extremely specific (96–98%) in patients with RA [26], but only 13.8% with SLE, 2.6% with SSc [27] and 7.5–9% with pSS [28], [29]. In our study, 21.4% of patients had at least one other autoimmune disease besides RA and these patients distributed similarly in the RA-only and RA-ILD groups. Further univariate and multivariate analyses revealed that these comorbidities did not affect the significant association of the anti-CCP2 positivity with RA-ILD in this population. Secondly, we stratified patients, according to the radiologic features by HRCT and we found that there was no significant difference in the anti-CCP2 positivity among these subgroups of patients,which was in consistent with a previous report [16]. Thirdly, MTX has been considered as the standard therapy of DMARDs for RA patients. Although MTX has been considered as a low toxic drug, MTX treatment of patients with pre-existing RA-ILD can cause fatal complication [30]. In our study, we did not observe that treatment with MTX resulted in a fatal outcome. There was no significant difference in the anti-CCP2 positivity between the patients receiving MTX treatment and those without MTX treatment. Thus, MTX treatment may not affect the levels of serum anti-CCP2 in RA patients. Finally, we did not observe that regular smoking was associated with RA-ILD in this population of RA patients, which was in consistent with previous reports [31].

The positivity of anti-CCP2 and RF is valuable for the diagnosis of RA. We found that the percentages of patients with positive anti-CCP2 and/or RF in the RA-ILD group were significantly higher than that in the RA-only group. Further analysis revealed that the percentages of the older patients with anti-CCP2 positivity in the RA-ILD group were significantly higher than that in the RA-only group. The univariate model of logistic regression analyses indicated that the different levels of serum anti-CCP2, but only high levels of RF were associated with ILD in this population of RA patients. Further adjusting other covariates revealed that the positivity of anti-CCP2 was significantly correlated with ILD in RA patients. Our data were in consistent with recent reports in France [18] and Greece [12] and similar to that in Japan [32]. However, there was no significant difference in the positivity of anti-CCP2 between the RA patients with and without ILD in another Japanese population [16] and a Korean population [17]. The difference among these studies may come from difference in patient population, disease definition or methodology for detecting clinical parameters. The variable sensitivity and specificity of the methods for anti-CCP2 detection may also affect its value in evaluating the association with ILD. The significantly higher positivity in the RA-ILD patients indicated that the positivity of anti-CCP2 may be a good biomarker for the diagnosis of ILD in RA patients.

We recognized that our study had limitations. We could not exclude the potential selection bias in our patients although individual patients were diagnosed as having ILD, according to the available diagnostic criteria [33]. Previous studies have estimated the prevalence of ILD in RA patients being about 3.7–33.0% in those without significant abnormal chest X-ray or respiratory symptoms [34]–[36]. It is possible that we may underestimate the prevalence of ILD in RA patients. In addition, we did exclude individual patients without complete medical records and those developed ILD prior to their RA diagnosis. Moreover, we had no complete medical records of all participants so that we were unable to evaluate whether a delay in treatment of patients with MTX and DMADRs can be a risk factor of ILD in RA patients. Finally, the small sample size in our study may interfere with some results, such as the lack of significant difference in the positivity of anti-CCP2 in different subtypes of ILD in this population. Furthermore, small sample size can lead to a false positive result and overestimate the magnitude of an association, particularly in a situation with multiple confounding variants. Therefore, further study in a bigger population is warranted to validate the value of anti-CCP2 positivity in evaluating the prognosis of ILD in RA patients.

In summary, we found significantly higher positivity of anti-CCP2 in RA patients with ILD than that in the RA alone patients. Our findings indicated that the positivity of anti-CCP2 is associated with ILD in RA patients.
